# A Novel Hybrid Surgical Technique for Large Renal Masses—Hybrid Technique of Laparoscopic-Assisted Open Radical Nephrectomy

**DOI:** 10.5152/tud.2025.24090

**Published:** 2025-07-29

**Authors:** Bhavana Chowdary, Arjun Nagaraj, Dhanshekar Nalluru, Nagaraj Harohalli Krishnareddy, Koustubh Gaonkar, Abhishek Reddy, Dinesh Anne, Murali Velagapudi

**Affiliations:** Sapthagiri Institute of Medical Sciences and Research Centre, Karnataka, India

**Keywords:** Large renal mass, nephrectomy, kidney cancer, hybrid technique

## Abstract

**Objective:**

The complex patient dynamic in India leads to diverse presentations of renal cell carcinoma, ranging from incidental small renal masses to large palpable renal masses. Minimally invasive surgical approaches pose challenges for patients with large renal masses (≥7 cm), prompting many urologists to opt for open radical nephrectomy.

However, open surgery is associated with higher morbidity due to prolonged visceral exposure and increased intraoperative bleeding. Large renal masses often exhibit significant neovascularity, complicating dissection and elevating intraoperative blood loss risk. This problem led the authors to devise a novel hybrid technique of laparoscopic-assisted open radical nephrectomy (HLO-RN), which helps to decrease morbidity.

In this article, the authors discuss this novel hybrid technique incorporating the benefits of both open and laparoscopic approaches (HLO-RN).

**Methods:**

The authorsconducted an observational study to report the authors’ HLO-RN, in 5 patients with large renal masses (≥7 cm) suggestive of renal cell carcinoma. Patients with morbid obesity (BMI ≥40) were excluded. The hybrid technique involves initial laparoscopy, followed by open flank incision after vessel clipping. Conversion-to-open procedure can be adjusted based on intraoperative conditions. In patients with inferior vena cava (IVC) thrombus extension, conversion-to-open procedure is made after sequential clamping of renal vessels and IVC with or without hepatic mobilization.

All the patients were followed for 6 months. Various parameters including patient characteristics, renal mass characteristics, staging, mean duration of laparoscopic and open procedures, intraoperative and postoperative complications, and duration of hospital stay and duration to return to normal activity were recorded.

**Results:**

Five patients (mean age: 61.2 years) were included, with 2 presenting with venous tumor thrombus extension. The mean renal mass size was 10.7 cm (range: 7.8-14 cm). One patient with IVC Level I thrombus required open conversion after sequential vessel clamping due to significant neovascularity. Partial laparoscopic mobilization was feasible in the remaining patients.

Procedure durations averaged 35.8 minutes (laparoscopic) and 35.6 minutes (open). All procedures were performed by the same senior urologist team. Patients were extubated immediately post-surgery, with one requiring intensive care unit admission for 1 day. No surgical site infections or major postoperative complications occurred. The drop in hemoglobin in this study was 0.84 g/dL.

**Conclusion:**

The authors’ novel HLO-RN technique is a practical and feasible approach for large renal masses, including those with IVC thrombus extension. By reducing intraoperative blood loss and open procedure duration, this hybrid technique significantly decreases perioperative morbidity.

## Introduction

The incidental finding of renal masses on the imaging performed for other indications led to an increased incidence of small renal masses.^1^ There is a unique epidemiology in the Indian patient population.^2^ This complex patient dynamic leads to unique presentations of various pathologies including renal cell carcinoma. Consequently, the authors have observed a rise in both small renal masses and large renal masses presenting with palpable mass

Kidney cancer and renal cell carcinoma are commonly used synonymously although renal cell carcinoma is the most common form of kidney cancer (9 out of 10 kidney cancers are renal cell carcinomas).^3^ Radical nephrectomy (RN) has been the traditional mainstay treatment option for localized and locally advanced renal cancers. However, patients with large renal masses have an inherent difficulty with minimally invasive surgical approaches like laparoscopic or robotic-assisted or robotic radical nephrectomies. Hence, many Urologists opt for open RN for renal masses ≥7 cm. The open approach is proven to be of higher morbidity when compared to minimally invasive alternatives due to varied reasons like increased bleeding, prolonged exposure of viscera to external environment, wound complications, and delayed recovery. The patients presenting with such advanced disease often have other comorbidities as well, augmenting the intraoperative and perioperative risk and complications.

Most of the morbidity associated with an open surgery comes from increased bleeding, prolonged environmental exposure to internal organs, wound complications, and delayed recovery. This in turn causes a higher risk of fluid loss, dehydration, increased need for intraoperative fluids, cardiac overload, risk of infection, and so forth. Moreover, increased exposure and handling of the bowel during the open procedure leads to prolonged postoperative ileus.

To address these challenges, the authors devised a novel method to decrease the amount of bleeding and reduce the duration of the open procedure by incorporating prior laparoscopic dissection of renal vessels. In the authors’ opinion, this HLO-RN helps reduce the morbidity associated with the open procedure for large renal masses.

In this article, the authors discuss this novel hybrid technique incorporating the benefits of both open and laparoscopic approaches (HLO-RN).

## Material and Methods

The authors designed an observational study involving 5 patients to report the authors’ novel hybrid technique of RN. A written informed consent was obtained from all the study participants. Ethics committee approval was obtained from the ethics committee of Sapthagiri Institute of Medical Sciences and Research Centre institute with approval number- SIMS&RC/EC/01/2023--24 on 12-03-2024.

The authors included 5 patients with large renal mass, i.e., ≥7 cm suggestive of renal cell carcinoma in this study. Patients with morbid obesity (BMI ≥ 40) were excluded from the study. After obtaining informed consent and institutional ethical committee approval for the study, the 5 subjects are enrolled in this study to report the authors’ novel hybrid technique. The preoperative examination and workup were performed as per the standard care protocols. The hybrid technique involves performing a laparoscopic clipping of renal vessels before opening the abdomen during RN.

All the patients were followed for 6 months. Various parameters including patient characteristics, renal mass characteristics, staging, mean duration of laparoscopic and open procedures, intraoperative and postoperative complications, duration of hospital stay, a requirement for intensive care unit admission, and duration to return to work/normal activity were recorded.

### The Technique

**Figure 1:**
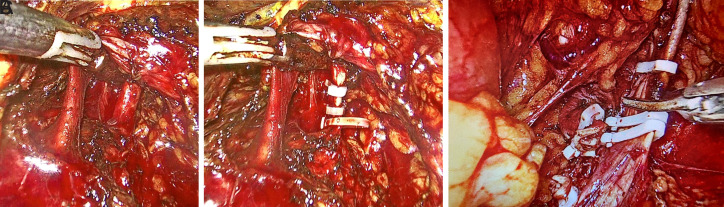
(A) Laparoscopic dissection of renal vessels. (B) Renal artery clipped. (C) Renal vein clipped.

General anesthesia was administered to all patients. Each patient was placed in a contralateral flank position with the ipsilateral lower limb extended and the contralateral lower limb flexed. The ipsilateral upper limb was extended in less than a 90-degree angle away from the operative field. The contralateral upper limb was then strapped to the torso. Two 10 mm and two 5 mm laparoscopic ports were placed as depicted in Figure 1 after creating pneumoperitoneum. Ureter and renal vessels are identified as per the standard procedure. The renal artery is clipped and cut first, followed by the renal vein. The ureter was also clipped and cut. Renal mass mobilization is done as much as possible.

Subsequently, conversion to an open procedure was made by creating a subcostal flank incision involving the midclavicular subcostal laparoscopic port. Access to the kidney was gained and further mobilization of the kidney was completed. The specimen was extracted through the incision and sent for histopathological examination.

Adrain was placed through the other 5 mm laparoscopic port without making an extra incision. The camera accesses 10 mm port site, and the main open incision wound were closed in layers.

The exact point of conversion to the open procedure can be adjusted according to the patient’s intraoperative condition. In patients with increased neovascularization and adhesions, where laparoscopic dissection and mobilization are not possible, conversion to an open procedure was made after ligation of main renal vessels (Figure 1A-C). In patients with inferior vena cava (IVC) thrombus extension, conversion to open procedure was made after sequential clamping of renal vessels and IVC with or without hepatic mobilization.

## Results

Five patients with suspected renal cell carcinoma of size exceeding 7 cm in size were included in the study. Four of the study subjects were male and 1 female, with a mean age of 61.2 years. Two subjects had the venous extension of the tumor thrombus. Both of them classified as level I thrombus i.e., extension into IVC <2 cm of renal vein level. Three of the patients presented with a palpable mass per abdomen, while the other 2 patients presented with non-specific symptoms. Imaging revealed a mean size of the renal mass lesion to be 10.7 cm ranging from 7.8 cm to 14 cm in maximum dimension. The mean ECOG performance status of the study subjects was 0.8. The patient characteristics are summarized in Table 1.

One subject with IVC level I thrombus extension displayed a very significant neovascularization around the lesion. Hence, the authors opted for open conversion after sequential clamping of vessels. In the remaining patients, despite the neovascularization, the authors could perform laparoscopic mobilization of renal mass partially.

A subcostal flank incision was employed in all the subjects. The mean duration of the laparoscopic part of the procedure was 35.8 minutes (29-52 minutes) The mean duration of the open part of the procedure was 35.6 minutes (26-40 minutes). All the procedures were performed by the same senior urologist team. None of the patients required intraoperative blood transfusion. None experienced surgical site infections or major postoperative complications. All the subjects were extubated immediately following surgery. One patient with a poor performance index required intensive care unit (ICU) admission for 1 day postoperatively.

The mean preoperative serum creatinine of the subjects was 0.96 mg/dL. Postoperative assessments on POD7 and at 6 months yielded mean serum creatinine levels of 0.98 mg/dL and 0.96 mg/dL, respectively. The mean duration of hospital stay was 5.2 days (4-7 days) and the mean time to return to work/normal activity was 15.8 days (12-19 days). All the study subjects remained under regular follow-up at the 6-month mark.

## Discussion

Radical nephrectomy is the standard of care for many patients with renal cell carcinomas.^4^ It includes excision of the kidney along with Gerota’s facia with/without adrenalectomy. Partial nephrectomy has been developed for small renal masses and is proven to be oncologically non-inferior to RN in specific patient populations.^5^ Although there is a rise in the presentation of small renal masses, the authors still face patients presenting with complex large renal masses. This delayed presentation may be attributed the indolent nature of the disease and partly due to a lack of awareness and access to health care in certain patient populations.

Various approaches to RN have been developed over the period, ranging from the standard open approach to the technologically advanced and minimally invasive robotic approaches.^6^The authors discuss the unique features of each approach below.

### Open Radical Nephrectomy

Open radical nephrectomy has been the standard approach for large renal malignancies.^7^ It is often performed through a transperitoneal approach although retroperitoneal access is also practiced. The open approach provides the advantage of space and ease of access and mobilization in large renal masses. For the specimen to be extracted as a whole for histopathological examination, a significant open incision is to be made in other minimal access RNs as well. The major disadvantage encountered is the perioperative morbidity associated with the open approach.

### Laparoscopic Radical Nephrectomy

The laparoscopic approach is the most commonly preferred and performed approach for renal masses, particularly small renal masses. Laparoscopic partial nephrectomy is the current procedure of choice for small renal masses. The advent of laparoscopy significantly reduced the perioperative morbidity and complication rates compared to open RN. Various studies show that there is no significant difference in the OS and CSS between open and laparoscopic RNs.^8^

### Hand-Assisted Laparoscopic Radical Nephrectomy

Hand-assisted approaches were developed to address the inherent difficulties faced during laparoscopy such as tactile feedback, long learning curve, and limited range of motion.^9^ This approach initally met with a huge response as it provides the best of both worlds, laparoscopy, and open approaches. However, this approach was largely abandoned with the increase in laparoscopic skills and expertise among urologists. Hand-assisting ports, which are a basic necessity for this approach, are also an added expense.

### Robotic Radical Nephrectomy and Robotic-Assisted Laparoscopic Radical Nephrectomy

All of the procedures which can be performed laparoscopically are being performed with better outcomes using a robotic approach. Radical nephrectomy is no exception. The expenses of the robotic approach or robotic-assisted laparoscopic approach are still a major hurdle in many countries. The number of urologists having expertise with the robotic approach is also limited in the present day. Hence, in specific patients with the availability of robotic services, robotic RN is a viable option.^10^

### Hybrid Laparoscopic-Assisted Open Radical Nephrectomy 

The majority of the perioperative morbidity in open RN is attributable to increased bleeding, prolonged environmental exposure to internal organs, and increased fluid loss and wound complications. In the authors’ novel hybrid technique, laparoscopic ligation of renal vessels reduces the bleeding and duration of the open part of the surgery.

The decreased intraoperative bleeding in this HLO-RN can be attributable to the advantage of enhanced magnification and better visibility, which lead to much finer dissection, easier identification, and ligation of renal vessels during laparoscopy. Although the overall operative duration is slightly increased in HLO-RN, there is a very significant decrease in intraoperative blood loss and open procedural duration, all of which lead to lesser morbidity and better outcomes.

With an increasing number of laparoscopic surgeries performed recently, urologists are more often exposed to laparoscopy than open procedures during the training years and early practice. This hybrid technique also serves best to such new-generation urologists who are much more familiar with laparoscopy.

Laparoscopy provides direct access to renal vessels and helps in easier identification and ligation even in patients with huge renal masses. Once, renal vessels are ligated and the ureter is identified, renal mobilization and specimen extraction can be done through a relatively smaller subcostal flank incision. Whereas a direct Open approach would require a larger rib-cutting flank incision or anterior midline incision or Chevron incision.

The increase in the size of the incision is associated with an increase in postoperative pain, risk of incisional hernia, surgical site infections, bleeding, prolonged hospital stay, delay in return to work, and higher overall postoperative morbidity. The duration of the open procedure is also proven to increase the risk of surgical site infections, deep vein thrombosis, increased ICU requirement, prolonged hospital stay, and postoperative delayed recovery. It is also associated with increased fluid loss intraoperatively, increased anesthesia-related complications like postoperative delayed extubation, and ventilator-associated infections.

Two out of 5 patients in this study also had an IVC thrombus. In these patients, performing laparoscopy before opening the abdomen provides the added advantage of dissecting out the contralateral renal vein and hepatic mobilization which can be performed laparoscopically, which otherwise would require an extensive open incision and increased duration in open surgery. In patients with IVC thrombus, this hybrid technique is especially useful since it significantly decreases the size of the open incision and operative duration.

The authors observed there was no change in renal parameters (serum creatinine) postoperatively except a insignificant rise of 0.1 mg/dL @ 1week postoperatively in one of the subjects. But at 6 months follow-up, there was no change in renal parameters compared to preoperative values in all the 5 subjects. This signifies there was no additional acute or chronic renal insult due to this hybrid technique, which may be expected because of the pneumoperitoneum during laparoscopic part of this hybrid technique.

Large renal masses with significant neovascularity are difficult to handle by completely laparoscopic RN. In such cases, surgeons tend to choose standard open RN if identified preoperatively or Lap converted to open RN (without handling renal vessels laparoscopically) if identified intraoperatively. In such patients, the authors’ hybrid technique comes to help. The vital difference is that laparoscopic clamping of renal vessels will be done laparoscopically. This helps in reduced intraoperative bleeding and decreased duration of open part of the surgery. Currently open RN is preferred in special circumstances such as complex large masses, cytoreductive nephrectomies and in patients with IVC thrombus.^7^ In all such cases, the authors believe that the authors’ hybrid technique will be better helpful to the patient.

The drop in hemoglobin in this study was 0.84 g/dL. The average blood loss as reported by Jeon et al was 604 mL in open RN.^11^ The mean duration of open part of the authors’ technique was 35.6 minutes, whereas completely open RN with IVC thrombus takes an average of 201 minutes.^11^ But the total duration of the authors’ hybrid technique including laparoscopic part was comparable to open RN.

The size of the incision,duration of the open procedure and intraoperative blood loss can be significantly reduced using the authors’ novel hybrid technique (HLO-RN). These factors are significantly capable of reducing perioperative morbidity and complications. This hybrid technique is also in line with interests of the present-day urologists with greater exposure to laparoscopy.

### Limitations

Our study’s main limitations are its small sample size and lack of a control arm. The outcomes of this novel hybrid technique can be clearly demonstrated when compared to standard completely open nephrectomy. A multicenter study with a larger sample size and a control arm of open RN would better assess the efficacy of HLO-Rn (hybrid technique).

The hybrid technique of RN, incorporating laparoscopy before open surgery, offers significant practical benefits and is a viable approach for managing large renal masses. Notably, this novel hybrid technique is particularly advantageous in patients with IVC thrombus extension. By combining the benefits of laparoscopy and open surgery, this hybrid technique substantially reduces perioperative morbidity through: reduced intraoperative blood loss, minimized incision size, and shortened duration of the open procedure.

This innovative approach enhances patient outcomes, making it a valuable addition to the surgical management of complex renal malignancies.

### Future Recommendations

Randomized control trials are recommended in future to quantify the benefit of this novel hybrid approach over open RN for large renal masses.

## Figures and Tables

**Table 1.** Patient Characteristics

Characteristic	Patient 1	Patient 2	Patient 3	Patient 4	Patient 5
Age (years)	54	72	57	67	56
Sex	F	M	M	M	M
ECOG performance status	1	3	0	0	0
Comorbidities	Hypertension	Hypertension, IHD	–	–	Hypertension
Size of lesion	12.2	7.8 cm	14 cm	10 cm	9.5 cm
Clinical staging					
Tumor thrombus	Level I	–	–	Level I	–
Duration of laparoscopy	52 minutes	24 minutes	35 minutes	39 minutes	29 minutes
Duration of open procedure	34 minutes	26 minutes	40 minutes	36 minutes	32 minutes
ICU admission	–	1 day	–	–	–
Major perioperative complications	–	–	–	–	–
Hospital stay	5 days	10 days	4 days	7 days	5 days
Drop in Hb (g/dL)	0.5	1.2	0.8	1.0	0.7
Mortality at 6 months	–	–	–	–	–
Margins for tumor on HPE	Negative	Negative	Negative	Negative	Negative
Preoperative serum creatinine (mg/dL)	1.2	1.0	0.7	0.9	1.0
Postoperative serum creatinine @ 1 week	1.2	1.1	0.7	0.9	1.0
Serum creatinine @ 6 months	1.2	1.0	0.7	0.9	1.0

Hb, hemoglobin; ICU, intensive care unit.

## Data Availability

The data that support the findings of this study are available on request from the corresponding author.
